# Network-Level Connectivity Dynamics of Movie Watching in 6-Year-Old Children

**DOI:** 10.3389/fnhum.2015.00631

**Published:** 2015-11-23

**Authors:** Robert W. Emerson, Sarah J. Short, Weili Lin, John H. Gilmore, Wei Gao

**Affiliations:** ^1^Department of Radiology and Biomedical Research Imaging Center, University of North CarolinaChapel Hill, NC, USA; ^2^Carolina Institute for Developmental Disabilities, University of North CarolinaChapel Hill, NC, USA; ^3^Department of Psychiatry, University of North CarolinaChapel Hill, NC, USA; ^4^Biomedical Imaging Research Institute, Department of Biomedical Sciences and Academic Imaging, Cedars-Sinai Medical CenterLos Angeles, CA, USA

**Keywords:** resting state functional connectivity, naturalistic stimuli, young children, functional network of the brain, developmental cognitive neuroscience

## Abstract

Better understanding of the developing brain’s functional mechanisms is critical for improving diagnosis and treatment of different developmental disorders. Particularly, characterizing how the developing brain dynamically reorganizes during different cognitive states may offer novel insight into the neuronal mechanisms of cognitive deficits. Imaging the brain during naturalistic conditions, like movie watching, provides a highly practical way to study young children’s developing functional brain systems. In this study we compared the network-level functional organization of 6-year-old children while they were at rest with their functional connectivity as they watched short video clips. We employed both a data-driven independent component analysis (ICA) approach and a hypothesis-driven seed-based analysis to identify changes in network-level functional interactions during the shift from resting to video watching. Our ICA results showed that naturally watching a movie elicits significant changes in the functional connectivity between the visual system and the dorsal attention network when compared to rest (*t*_(32)_ = 5.02, *p* = 0.0001). More interestingly, children showed an immature, but qualitatively adult-like, pattern of reorganization among three of the brain’s higher-order networks (frontal control, default-mode and dorsal attention). For both ICA and seed-based approaches, we observed a decrease in the frontal network’s correlation with the dorsal attention network (ICA: *t*_(32)_ = −2.46, *p* = 0.02; Seed-based: *t*_(32)_ = −1.62, *p* =0.12) and an increase in its connectivity with the default mode network (ICA: *t*_(32)_ = 2.84, *p* = 0.008; Seed-based: *t*_(32)_ = 2.28, *p* =0.03), which is highly consistent with the pattern observed in adults. These results offer improved understanding of the developing brain’s dynamic network-level interaction patterns during the transition between different brain states and call for further studies to examine potential alterations to such dynamic patterns in different developmental disorders.

## Introduction

Early school age is a particularly important developmental phase when complex brain functions are rapidly maturing, concurrent with children beginning their formal education. Studies aiming to unveil the mechanisms underlying normal human functional development are critical for better understanding and treatment of different developmental disorders. Functional connectivity studies based on naturalistic designs, such as movie watching, represent a unique opportunity for delineating functional brain mechanisms during a developmental period when task-based fMRI studies are not a viable option (Bartels and Zeki, 2005; Golland et al., 2007; Hasson et al., 2008, 2010; Emerson and Cantlon, 2012; Cantlon and Li, 2013). These types of studies provide a way to examine how the brain’s network-level interactions differ between states, such as being at rest, watching a movie, or performing a task, while offering a feasible method to study functional brain development. Understanding the development of the brain’s typical functional interactions represents an emerging emphasis for functional brain imaging research and may aid our understanding of both the brain’s intrinsic functional structure and how this structure reorganizes to process various types of information.

In the mature adult brain, three higher-order networks are likely involved in the transition from rest to naturalistic stimuli conditions. First, the default mode network, which is commonly associated with internally directed cognitions (Gusnard et al., 2001; Buckner and Vincent, 2007; Mason et al., 2007; Buckner et al., 2008; Andrews-Hanna et al., 2010) and is reportedly activated during movie watching conditions (Iacoboni et al., 2004; Golland et al., 2007). This network commonly involves regions in parietal cortex (precuneus and posterior cingulate), bilateral inferior–lateral–parietal and ventromedial frontal cortex. Second, the dorsal attention network, which is primarily activated during goal-directed activities that require focused attention (Corbetta and Shulman, 2002; Fox et al., 2005, 2006). This network commonly involves regions in the frontal eye fields, ventral premotor cortex, superior parietal lobule, intraparietal sulcus, and motion-sensitive middle temporal area. These two networks are spatially distinct and likely interact differentially with other higher-order networks during uncontrolled rest vs. movie watching conditions. For example, compared to rest, the visual system is likely to weaken its connection with the dorsal attention network while passively viewing a movie but strengthen its connection to this network if involved in strenuous visual search task. Third, the fronto-parietal control network is spatially interposed between these two previously defined networks and covers regions identified as supporting cognitive control and decision-making processes including lateral prefrontal cortex, anterior cingulate cortex, and inferior parietal lobule (Vincent et al., 2008). The fronto-parietal control network shows enhanced connectivity with the default network but reduced interaction with the dorsal attention network during movie watching compared to resting conditions. Research examining these interactions in adults indicates that the fronto-parietal control network provides a mediating role between the “competing” default and dorsal attention networks (Gao and Lin, 2012; Spreng et al., 2013; Elton and Gao, 2014). In other words, the fronto-parietal control network may play a role in guiding how the default and dorsal attention networks interact with each other as well as other functional networks. Taken together, research with adults indicates that the dynamic interaction between these three higher-order networks forms a functional set and that their interactions in the resting brain differ from their interactions during movie watching. However, in order to understand the developing brain’s dynamic reorganization mechanisms, these interactions critically need to be investigated in school-aged children.

In this study, we sought to delineate the changing functional connectivity of 6-year-old children’s network-level interaction patterns between rest and naturalistic movie watching by conducting functional magnetic resonance imaging (fMRI) scans during each of the two conditions. We tested the differences between these conditions in two steps. First, we performed a data-driven whole brain analysis that identified canonical functional networks and tested the changes between the conditions. Second, we used established regions of interest to perform a seed-based analysis identical to a previous analysis in adults (Gao and Lin, 2012). We predicted that school-age children’s pattern of network-level interactions would be similar to adults, but the magnitude of their functional interactions would be immature. Specifically, we predicted that the frontal control network should show an increase in connectivity with the default network but a decrease in connectivity with the dorsal attention network during movie viewing when compared with rest. Because of the increase in the complexity of visual stimulation, we also expected that the visual network would decrease its connectivity with the dorsal attention network and increase its connectivity with the default network during passive viewing.

## Materials and Methods

### Subjects

Thirty-three typically developing 6-year-old children were included in final analysis of this study (17 females and 16 males; mean age = 6.10 ± 0.11 years). All participants were healthy, with no history of neurological impairments or abnormalities and no parental history of major psychiatric illness. Participants were initially recruited for a larger longitudinal study examining brain development from birth through early childhood in typical and high-risk children (Li et al., 2014). Thus, any participants at genetic high-risk for schizophrenia or bipolar disorder, based on maternal diagnosis, were excluded from this study. All guidelines and requirements of the University of North Carolina’s Research Subjects Review Board were followed for participant recruitment and experimental procedures.

### Stimuli

For the resting state fMRI (rsfMRI) scan, children were instructed to keep their eyes open while a white fixation cross was presented in the middle of a black screen. The second connectivity scan was acquired while children viewed an age appropriate movie of their choice. Each scan lasted approximately 5 min and the resting state scan was always acquired first at the start of the imaging session.

### Image Acquisition

All images were acquired using a 3-T MR scanner (Siemens Medical Systems, Erlangen, Germany) housed in the Biomedical Research Imaging Center (BRIC). All fMRI data was acquired using a T2*-weighted EPI sequence: time repetition (TR) = 2 s, time echo (TE) = 32 ms, 33 slices, voxel size of 4 × 4 × 4 mm^3^. One hundred and fifty volumes were acquired in 5 min. In order to provide anatomical reference, structural images were acquired using a 3D Magnetization Prepared Rapid Acquisition Gradient-recalled Echo sequence (TR = 1820 ms, TE = 4.38 ms, in-version time = 1100 ms), with a voxel size of 1 × 1 × 1 mm^3^. To reduce motion children’s heads were secured with foam padding.

### Preprocessing

Functional data were preprocessed using FMRIB’s Software Libraries (FSL, v 4.1.9; Smith et al., 2004). The preprocessing steps included discarding the first 10 volumes, slice timing correction, motion correction, high-pass (>0.01 Hz) and low-pass filtering (<0.08 Hz). Mean signal from white matter, cerebrospinal fluid, whole brain, and six motion parameters were removed using linear regression. Spatial smoothing was applied with a Gaussian kernel of 6 mm full width at half maximum. In order to further reduce the effect of motion on functional connectivity measures, the “scrubbing” approach of controlling the global measure of signal change (0.5%) and frame-wise displacement (0.5 mm) was carried out as proposed by Power et al. (2012). Subjects with more than one-third of volumes (i.e., 50 volumes) removed from the scrubbing procedure were excluded from subsequent analyses. In total, three subjects were removed, leaving the final 33 subjects for further analysis. For each subject and session, after an initial rigid alignment between functional data and the T1 high-resolution structural images, a nonlinear transformation field was obtained from the individual T1 images to the MNI template using FSL. The combined transformation field was used to warp the preprocessed rsfMRI data to the template.

### Network Analyses

**Figure 1:**
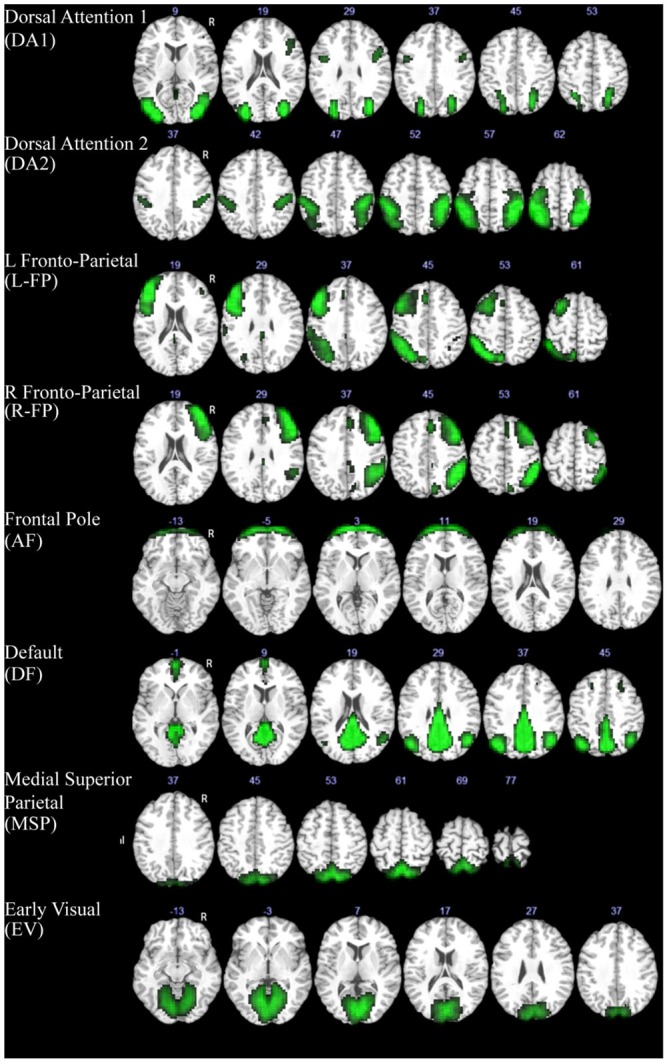
**Components extracted with ICA that showed a high spatial correlation with previously defined resting-state networks (Smith et al., 2009).** The MNI z coordinate value is listed above each image for reference.

Group-level independent component analysis (ICA) was performed on all rsfMRI data using GIFT v2.0 toolbox for Matlab (Calhoun et al., 2001). The 20 extracted group-level spatial ICA maps of independent resting-state networks (RSNs) were scaled to *z*-scores with a threshold of *z* > 3 (corresponding to a significance level of *p* < 0.05). These component maps were then visually inspected and labeled based on the spatial patterns in reference to known anatomical and functional locations (Tzourio-Mazoyer et al., 2002). Components that captured motion artifacts were removed. Subject-specific spatial maps and time courses were estimated for each remaining component and were then back-reconstructed using GIFT (Calhoun et al., 2001). We further identified a subset of components that matched one of the brain’s 10 canonical RSNs (Smith et al., 2009) and subsequent analysis were done on those components that are related to movie watching (i.e., the default mode network, the dorsal attention network, the frontoparietal network, and the visual networks). To ensure a good match, each component was spatially correlated with network templates and a threshold of *r* > 0.40 was used to identify corresponding components. In total there were eight identified components that met these criteria and were used for further analysis (Figure 1).

Individual whole brain spatial maps for each subject were compared between resting and movie watching using paired *t*-tests for each included component. Using each subjects’ back-reconstructed component time course, pairwise correlations were calculated between each component to assess network-level functional connectivity. Paired *t*-tests were then performed between resting and movie watching data for each of the network-level connections. Within-network connectivity was evaluated based on the back-reconstructed spatial map of each ICA network for each subject. Specifically, voxel-wise comparisons were done using paired *t*-test based on the individual spatial maps to test whether there were any significant differences in within-network connectivity. The false discovery rate (FDR; Benjamini and Yekutieli, 2001) method was used to correct for multiple comparisons and significant connections were defined at *q* < 0.05 after FDR correction.

**Figure 2:**
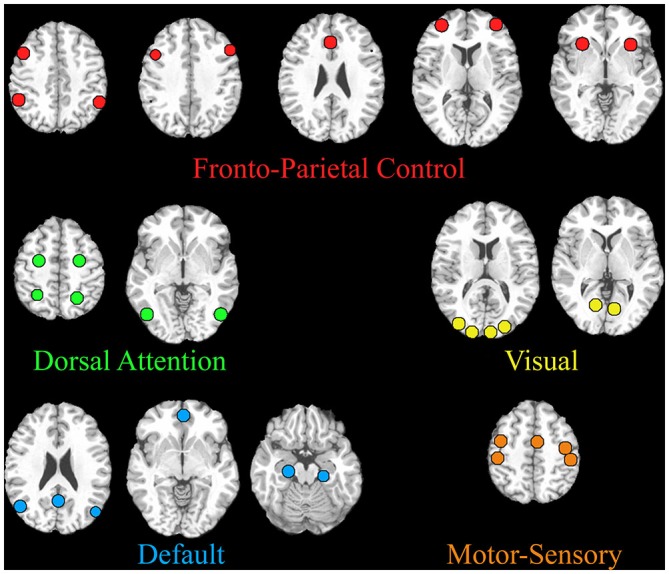
**Regions included in the seed-based network analysis are shown.** The MNI coordinates are listed in Table 1.

A seed-based analysis was also performed with network seeds that were previously tested in adults during a similar task (Gao and Lin, 2012). These network seeds are shown in Figure 2 and Table 1. Specifically, we tested the interconnectivity of the visual, dorsal attention, default mode, and fronto-parietal networks. A 5 mm sphere was created around each of the 32 coordinates and the average time course was extracted for each subject. For each individual, time courses were correlated between each network seed. After Fisher’s *z* transform and averaging across all subjects, group mean matrices were obtained for each pair of seeds during resting and movie watching states and then used to test the differences between both states. Each network’s connectivity was tested using a one sample *t*-test from 0 to identify significant positive/negative interactions. For between-network comparison, the interactions between two networks were averaged for each subject and then compared between movie watching and resting states using paired *t*-tests. Similarly, for within-network comparisons, the correlation between all nodes of a network were averaged for each subject and compared across states using paired *t*-tests. *P* < 0.05 after FDR correction was considered significant.

**Table 1:** **MNI coordinates for the regions of interest within the five predefined networks in adults**.

Dorsal attention	lMTþ: (−45, −69, −2)
	rMTþ: (50, −69, −3)
	lIPS: (−27, −52, 57)
	rIPS: (24, −56, 55)
	lFEF: (−25, −8, 50)
	rFEF: (27, −8, 50)
Fronto-parietal control	laPFC: (−36, 57, 9)
	raPFC: (34, 52, 10)
	ACC: (3, 31, 27)
	laIPL: (−52, −49, 47)
	raIPL: (52, −46, 46)
	ldlPFC: (−50, 20, 34)
	rdlPFC: (46, 14, 43)
	lINS: (−31, 21, −1)
	rINS: (31, 22, −2)
Default mode	lHF: (−21, −15, −14)
	rHF: (24, −19, −21)
	vmPFC: (0, 51, −7)
	PCC: (1, −55, 17)
	lpIPL: (−47, −71, 29)
	rpIPL: (50, −64, 27)
Visual	lCal: (−8, −72, 4)
	rCal: (16, −67, 5)
	lCS: (−5, −96, 12)
	rCS: (18, −96, 1)
	lLO: (−23, −89, 12)
	rLO: (37, −85, 13)

*These coordinates define the center of a 5 mm sphere used in the seed-based analysis. aPFC, anterior prefrontal cortex; dlPFC, dorsal lateral prefrontal cortex; ACC, anterior cingulate cortex; INS, insula; aIPL, anterior inferior parietal lobule; IPS, bilateral intra-parietal sulcus; FEF, frontal eye field; MTþ, middle temporal area; PCC, posterior cingulate cortex; MPFC, medial prefrontal cortex; pIPL, bilateral posterior inferior parietal lobule; HF, hippocampus formation; Cal, bilateral calcarine; CS, cuneus; LO, lateral occipital*.

## Results

### ICA Analysis

The ICA analysis produced a total of eight components that met the specified criterion and were included in further analyses. These networks included two dorsal attention components (DA1, DA2), bilateral frontal-parietal components (L-FP, R-FP), a default-mode network component (DMN), an anterior frontal control component (AF), a medial superior parietal component (MSP), and an early visual component (EV; Figure 1).

**Figure 3:**
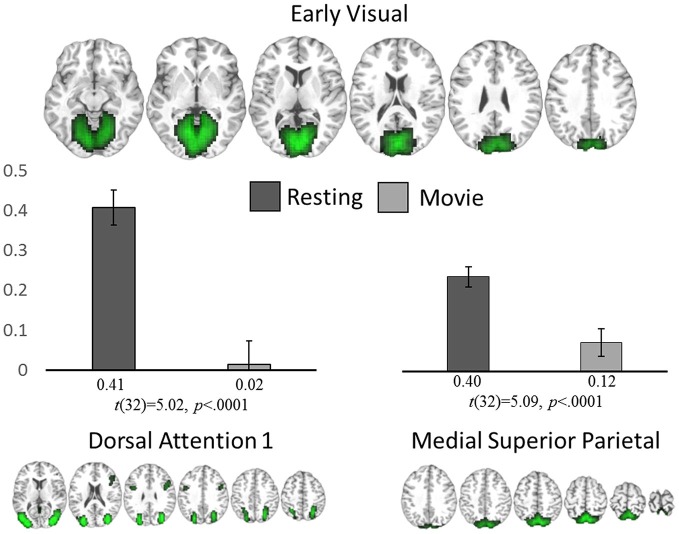
**ICA analysis.** Significant network-level changes between rest and movie watching using data-driven ICA analysis. The average connectivity value between the early visual network (top) to both the dorsal attention (bottom left) and medial superior parietal networks (bottom right) are shown for both resting state (dark gray) and movie watching (light gray).

Whole brain comparisons of the component spatial maps revealed no significant differences in within-network connectivity between rest and movie watching. However, two inter-network connections showed a significant difference; EV significantly reduced its connectivity with DA1 (*t*_(32)_ = −5.02, *p* = 0.0001) and MSP (*t*_(32)_ = −5.09, *p* = 0.0001) during movie watching (Figure 3) when compared with rest. At rest, the EV component was significantly positively correlated with both DA1 (*r* = 0.41, *p* = 0.01) and MSP (*r* = 0.40, *p* = 0.01) while during movie watching, it desynchronized with DA1 (*r* = 0.01, *p* = 0.83 in a one-sample *t*-test from 0) and maintained a reduced positive connectivity with MSP (*r* = 0.12, *p* = 0.03).

**Figure 4:**
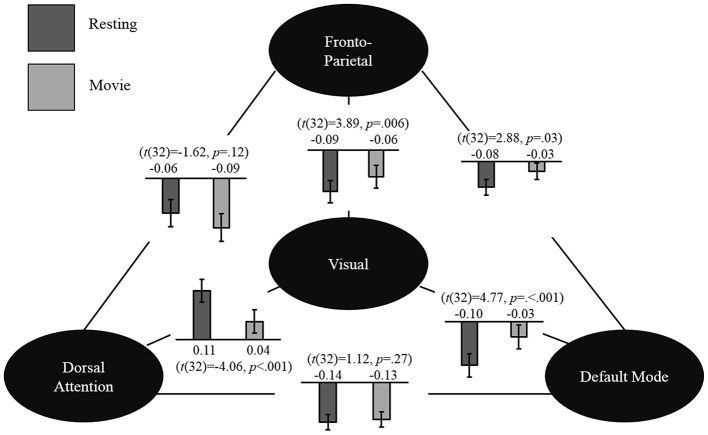
**Seed-based analysis.** Network-level changes between resting state and movie watching in the seed-based connectivity analysis are shown. The values shown represent the group mean connectivity for resting state (dark gray) and movie watching (light gray). Mean values and corresponding paired *t*-test are shown.

*Post hoc* analyses (described above) were carried out to test our hypothesis regarding the three higher order networks (i.e., DA1, DA2, DM, L-FP, R-FP and AF, Figure 4). While none of the differences in inter-network connections between rest and movie watching reached significance after FDR correction, there were some interesting trends. Specifically, AF decreased its correlation with DA1 (*t*_(32)_ = −2.46, *p* = 0.02) and increased its connectivity with DMN (*t*_(32)_ = 2.84, *p* = 0.008), which is highly consistent with the trend observed in adults. Additionally, AF also increased its connectivity with EV (*t*_(32)_ = 2.81, *p* = 0.008), and L-FP (*t*_(32)_ = 2.74, *p* = 0.009). Other state-related changes in connections between these networks did not show any uncorrected trends in significance (all *p* > 0.05). These results suggest that children share a similar but immature pattern of network-level functional reorganization during the transition from movie watching to rest when compared with adults.

#### Seed-Based Analysis

To further validate the observed pattern, we conducted seed-based analyses using previously defined network seeds in adults (Gao and Lin, 2012). As expected, the fronto-parietal network showed a trend of decreased connectivity with the dorsal attention network (*t*_(32)_ = −1.62, *p* = 0.12) and increased connectivity with the default mode network (*t*_(32)_ = 2.28, *p* = 0.03). Moreover, highly consistent with the ICA results reported above, the visual network significantly increased its connectivity with the default mode (*t*_(32)_ = 4.77, *p* < 0.001) and fronto-parietal networks (*t*_(32)_ = 3.89, *p* = 0.006), while significantly decreasing its connectivity with the dorsal attention network (*t*_(32)_ = −4.06, *p* = 0.001) during moving watching. Finally, the dorsal attention and default mode network were found to be anticorrelated during both rest (*r* = −0.14, *p* = 0.02) and movie viewing (*r* = −0.13, *p* = 0.03) and no significant difference was detected for this network-level interaction (*t*_(32)_ = 1.12, *p* = 0.27). The general pattern of results is displayed in Figure 4.

### Discussion

In this study, we aimed to delineate the dynamic reorganization pattern of the brain’s functional networks during the transition from rest to naturalistic movie watching in a cohort of healthy 6-year-old children. Our results revealed that movie watching in children elicits significant uncoupling between the visual input network and dorsal attention network, which is accompanied by an adult-like, but immature, reorganization pattern of the network-level interactions among three higher order networks: the dorsal attention, the default-mode, and the frontal control network. Characterizing typical functional connectivity dynamics in young children can provide insight into how the brain supports their growing set of cognitive skills and behaviors, and eventually may allow for objective identification of cognitive or functional delays and developmental abnormalities.

Many studies have found a relationship between disruptions of intrinsic functional networks (i.e., measured at rest) and atypical cognitive development in different neurodevelopmental disorders (Cao et al., 2009; Church et al., 2009; Vogel et al., 2010; Bray et al., 2011; Elton et al., 2014). However, to further extend our understanding of the neurobiological basis of developmental disorders with specific cognitive deficits, it is beneficial to examine the brain’s dynamic functional organization during the transition from resting to corresponding task states (Gao and Lin, 2012; Gao et al., 2013; Spreng et al., 2013; Elton and Gao, 2014, 2015). Presently, one of the biggest challenges in studying younger children’s functional development in the context of cognitive tasks is the fact that traditional task-based fMRI experimental designs often fail to engage young children, leading to a very low success rate. Collecting data during natural viewing conditions has the potential to usurp these challenges, representing a realistic and practical method to study the developing brain. Specifically, by experimentally controlling the content of the movies, researchers will be able to determine how the brain accesses particular types of information while under conditions that mirror those found in everyday experience, making it feasible to explore the functional organization of children’s minds before they enter school. Neuroimaging data from this critical time in development is currently extremely limited. Dynamic network-level functional connectivity analyses in young children using naturalistic stimuli, as done in this study, will benefit further research that aims to explore how individual differences in functional brain networks are related to different domains of cognitive functions.

Overall, our results suggest that the dynamic reorganization patterns of the brain’s major functional networks in early school-age children feature large changes related to the visual input network and an immature, but similar interaction pattern among three higher order cognitive networks. Specifically, we detected a significant reduction in functional connectivity between the visual input network and the dorsal attention network during movie watching. This pattern could reflect a shift in children’s visual processing away from the goal-directed focus of attention that is typically associated with the dorsal attention network when they watch movies. The dorsal attention network has been hypothesized to generate and maintain a goal-directed, top-down signal to selectively bias visual cortical activity (Gitelman et al., 1999; Hopfinger et al., 2000; Corbetta and Shulman, 2002) and is typically active during visual search, visuospatial cuing, and other goal-directed tasks that involve spatial attention. It’s possible that the movie watching state may incur substantial internally driven cognitive processes so the visual input network may become more coupled with the default-mode network. Indeed, a significant enhancement of the functional connectivity between the visual network and the default-mode network during movie watching was observed in our subsequent seed-based analyses. Therefore, our results indicated that there is likely a rebalancing of top-down control of the primary visual network during the transition from resting to movie watching, potentially reflecting a shift away from external, goal-directed search to internally driven processing.

For the dynamic interaction patterns among the dorsal attention, frontal control, and default-mode networks, our *post hoc* analyses revealed a qualitatively adult-like pattern. Specifically, the frontal control network shows up-regulated connectivity with the default mode network and down-regulated connectivity with the dorsal attention network (DA1) which is highly consistent with patterns observed in adults during similar movie watching state (Gao and Lin, 2012). This ICA-derived pattern is further confirmed with our seed-based hypothesis-driven analysis, supporting the robustness of this finding. However, the children’s network-level interactions among these three higher-order cognitive networks only show marginally significant changes between resting state and movie watching. With the bigger sample size employed in this study compared with other adult studies it is not likely that these statistically weaker changes are driven by a lack of power. Rather, these results suggest that the network-level dynamic interaction patterns observed in adults are immature in children.

In contrast to the immature connections observed in our ICA analysis, we consistently found a relatively adult-like anticorrelation pattern between the default-mode network and dorsal attention network in children during both rest and movie watching. This anticorrelated relationship represents a mechanism that is postulated to mediate the switch between internal and external processes (Corbetta and Shulman, 2002; Fox et al., 2005, 2006). One of our previous studies suggests that this relationship emerges during infancy (Gao et al., 2009, 2013) so it would likely be already established by 6 years of age. Given this adult-like interaction pattern between the default mode and dorsal attention networks, the immature reorganization pattern among the three higher-order networks observed in this study is likely driven by the protracted development of the frontal control network. By 6 years of age, the brain volume is already approximately 95% of the adult size. However, at this period in development the brain is undergoing a critical fine-tuning process along with forms of cellular maturation, such as myelination and synaptic pruning, allowing for further cognitive development, particularly in the frontal lobe (Rakic et al., 1994; Huttenlocher and Dabholkar, 1997; Amso and Casey, 2006; Petanjek et al., 2008). Behaviorally, higher-order executive control functions such as planning, cognitive flexibility, inhibitory control, and attention change dramatically from early childhood to young adulthood (Luna et al., 2004). Resting state fMRI studies have demonstrated a shift from diffuse to focal activation patterns and simultaneous pruning of local connectivity paired with strengthening of long-range connectivity from later childhood to late adolescence (Fair et al., 2009; Power et al., 2010; Uddin et al., 2010). Therefore, it is likely that the maturation of the functional connections of the frontal network underlies the continued development of children’s executive functions, particularly with their ability to regulate the processes governed by the dorsal attention and default networks. Further research linking individual differences in functional network organization to cognitive function is needed to support this hypothesis.

One limitation of the current study is that our subjects are watching different movies of their own choice, which limits specific interpretations of the observed functional connectivity changes. Future studies that utilize the same movie stimuli across subjects are warranted to validate the current findings. Further research is also needed to determine the effects that the immature relationship between the frontal control network and the dorsal attention and default networks have on children’s individual cognitive and behavioral profiles. Eventually such knowledge might allow researchers to assess children’s risk for various disorders, like ADHD, from a very young age.

## Conclusion

In the current study, we identified differences in the network-level functional connectivity during the naturalistic condition of movie watching compared to resting in a cohort of healthy 6-year-old children. Our results from both the data-driven ICA and hypothesis-driven seed-based analyses show that changes in children’s functional interactions during naturalistic movie watching are supported primarily by their visual system in tandem with their developing higher-order cognitive networks. Specifically, significant changes in connectivity between the visual and attention-related networks are observed which are accompanied by a maturing tri-network interaction patterns among the default-mode, dorsal attention, and frontal pole networks. By examining the dynamic functional connectivity changes between resting state and naturalistic movie watching in young children, this study provides novel insights into our understanding of how children process complex stimuli, revealing a developmental immaturity in the frontal lobe’s contribution to the interactions between three of the brain’s higher-order functional networks. Future studies using a similar design could provide a more advanced insight into how the brain’s functional networks, in the early years of life, are related to typical cognitive development and developmental disorders.

## Conflict of Interest Statement

The authors declare that the research was conducted in the absence of any commercial or financial relationships that could be construed as a potential conflict of interest.

## References

[B1] Amso D. Casey B. (2006). Beyond what develops when neuroimaging may inform how cognition changes with development. Curr. Dir. Psychol. Sci. 15, 24–29. 10.1111/j.0963-7214.2006.00400.x

[B2] Andrews-Hanna J. R. Reidler J. S. Sepulcre J. Poulin R. Buckner R. L. (2010). Functional-anatomic fractionation of the brain’s default network. Neuron 65, 550–562. 10.1016/j.neuron.2010.02.005 20188659 PMC2848443

[B3] Bartels A. Zeki S. (2005). Brain dynamics during natural viewing conditions-a new guide for mapping connectivity *in vivo*. Neuroimage 24, 339–349. 10.1016/j.neuroimage.2004.08.044 15627577

[B4] Benjamini Y. Yekutieli D. (2001). The control of the false discovery rate in multiple testing under dependency. Ann. Stat. 29, 1165–1188.

[B5] Bray S. Dunkin B. Hong D. S. Reiss A. L. (2011). Reduced functional connectivity during working memory in Turner syndrome. Cereb. Cortex 21, 2471–2481. 10.1093/cercor/bhr017 21441396 PMC3183420

[B6] Buckner R. L. Andrews-Hanna J. R. Schacter D. L. (2008). The brain’s default network. Ann. N Y Acad. Sci. 1124, 1–38. 10.1196/annals.1440.011 18400922

[B7] Buckner R. L. Vincent J. L. (2007). Unrest at rest: default activity and spontaneous network correlations. Neuroimage 37, 1091–1096; discussion 1097–1099. 10.1016/j.neuroimage.2007.01.010 17368915

[B8] Calhoun V. Adali T. Pearlson G. Pekar J. (2001). A method for making group inferences from functional MRI data using independent component analysis. Hum. Brain Mapp. 14, 140–151. 10.1002/hbm.1048 11559959 PMC6871952

[B9] Cantlon J. F. Li R. (2013). Neural activity during natural viewing of Sesame street statistically predicts test scores in early childhood. PLoS Biol. 11:e1001462. 10.1371/journal.pbio.1001462 23300385 PMC3536813

[B10] Cao X. Cao Q. Long X. Sun L. Sui M. Zhu C.. (2009). Abnormal resting-state functional connectivity patterns of the putamen in medication-naive children with attention deficit hyperactivity disorder. Brain Res. 1303, 195–206. 10.1016/j.brainres.2009.08.029 19699190

[B11] Church J. A. Fair D. A. Dosenbach N. U. Cohen A. L. Miezin F. M. Petersen S. E.. (2009). Control networks in pediatric Tourette syndrome show immature and anomalous patterns of functional connectivity. Brain 132(Pt. 1), 225–238. 10.1093/brain/awn223 18952678 PMC2638693

[B12] Corbetta M. Shulman G. L. (2002). Control of goal-directed and stimulus-driven attention in the brain. Nat. Rev. Neurosci. 3, 201–215. 10.1038/nrn755 11994752

[B15] Elton A. Alcauter S. Gao W. (2014). Network connectivity abnormality profile supports a categorical-dimensional hybrid model of ADHD. Hum. Brain Mapp. 35, 4531–4543. 10.1002/hbm.22492 24615988 PMC4213949

[B13] Elton A. Gao W. (2014). Divergent task-dependent functional connectivity of executive control and salience networks. Cortex 51, 56–66. 10.1016/j.cortex.2013.10.012 24315034

[B14] Elton A. Gao W. (2015). Task-related modulation of functional connectivity variability and its behavioral correlations. Hum. Brain Mapp. 36, 3260–3272. 10.1002/hbm.22847 26015070 PMC6869497

[B16] Emerson R. W. Cantlon J. F. (2012). Early math achievement and functional connectivity in the fronto-parietal network. Dev. Cogn. Neurosci. 2(Suppl. 1), S139–S151. 10.1016/j.dcn.2011.11.003 22682903 PMC3375498

[B17] Fair D. A. Cohen A. L. Power J. D. Dosenbach N. U. Church J. A. Miezin F. M.. (2009). Functional brain networks develop from a “local to distributed” organization. PLoS Comput. Biol. 5:e1000381. 10.1371/journal.pcbi.1000381 19412534 PMC2671306

[B18] Fox M. D. Corbetta M. Snyder A. Z. Vincent J. L. Raichle M. E. (2006). Spontaneous neuronal activity distinguishes human dorsal and ventral attention systems. Proc. Natl. Acad. Sci. U S A 103, 10046–10051. 10.1073/pnas.0604187103 16788060 PMC1480402

[B19] Fox M. D. Snyder A. Z. Vincent J. L. Corbetta M. Van Essen D. C. Raichle M. E. (2005). The human brain is intrinsically organized into dynamic, anticorrelated functional networks. Proc. Natl. Acad. Sci. U S A 102, 9673–9678. 10.1073/pnas.0504136102 15976020 PMC1157105

[B20] Gao W. Gilmore J. H. Shen D. Smith J. K. Zhu H. Lin W. (2013). The synchronization within and interaction between the default and dorsal attention networks in early infancy. Cereb. Cortex 23, 594–603. 10.1093/cercor/bhs043 22368080 PMC3563337

[B21] Gao W. Lin W. (2012). Frontal parietal control network regulates the anti-correlated default and dorsal attention networks. Hum. Brain Mapp. 33, 192–202. 10.1002/hbm.21204 21391263 PMC3131466

[B22] Gao W. Zhu H. Giovanello K. S. Smith J. K. Shen D. Gilmore J. H.. (2009). Evidence on the emergence of the brain’s default network from 2-week-old to 2-year-old healthy pediatric subjects. Proc. Natl. Acad. Sci. U S A 106, 6790–6795. 10.1073/pnas.0811221106 19351894 PMC2672537

[B23] Gitelman D. R. Nobre A. C. Parrish T. B. LaBar K. S. Kim Y.-H. Meyer J. R.. (1999). A large-scale distributed network for covert spatial attention further anatomical delineation based on stringent behavioral and cognitive controls. Brain 122, 1093–1106. 10.1093/brain/122.6.1093 10356062

[B24] Golland Y. Bentin S. Gelbard H. Benjamini Y. Heller R. Nir Y.. (2007). Extrinsic and intrinsic systems in the posterior cortex of the human brain revealed during natural sensory stimulation. Cereb. Cortex 17, 766–777. 10.1093/cercor/bhk030 16699080

[B25] Gusnard D. A. Akbudak E. Shulman G. L. Raichle M. E. (2001). Medial prefrontal cortex and self-referential mental activity: relation to a default mode of brain function. Proc. Natl. Acad. Sci. U S A 98, 4259–4264. 10.1073/pnas.071043098 11259662 PMC31213

[B26] Hasson U. Furman O. Clark D. Dudai Y. Davachi L. (2008). Enhanced intersubject correlations during movie viewing correlate with successful episodic encoding. Neuron 57, 452–462. 10.1016/j.neuron.2007.12.009 18255037 PMC2789242

[B27] Hasson U. Malach R. Heeger D. J. (2010). Reliability of cortical activity during natural stimulation. Trends Cogn. Sci. 14, 40–48. 10.1016/j.tics.2009.10.011 20004608 PMC2818432

[B28] Hopfinger J. B. Buonocore M. H. Mangun G. R. (2000). The neural mechanisms of top-down attentional control. Nat. Neurosci. 3, 284–291. 10.1038/72999 10700262

[B29] Huttenlocher P. R. Dabholkar A. S. (1997). Regional differences in synaptogenesis in human cerebral cortex. J. Comp. Neurol. 387, 167–178. 10.1002/(sici)1096-9861(19971020)387:2<167::aid-cne1>3.0.co;2-z 9336221

[B30] Iacoboni M. Lieberman M. D. Knowlton B. J. Molnar-Szakacs I. Moritz M. Throop C. J.. (2004). Watching social interactions produces dorsomedial prefrontal and medial parietal BOLD fMRI signal increases compared to a resting baseline. Neuroimage 21, 1167–1173. 10.1016/j.neuroimage.2003.11.013 15006683

[B31] Li G. Wang L. Shi F. Lyall A. E. Ahn M. Peng Z.. (2014). Cortical thickness and surface area in neonates at high risk for schizophrenia. Brain Struct. Funct. [Epub ahead of print]. 1–15. 10.1007/s00429-014-0917-3 25362539 PMC4452433

[B32] Luna B. Garver K. E. Urban T. A. Lazar N. A. Sweeney J. A. (2004). Maturation of cognitive processes from late childhood to adulthood. Child Dev. 75, 1357–1372. 10.1111/j.1467-8624.2004.00745.x 15369519

[B33] Mason M. F. Norton M. I. Van Horn J. D. Wegner D. M. Grafton S. T. Macrae C. N. (2007). Wandering minds: the default network and stimulus-independent thought. Science 315, 393–395. 10.1126/science.1131295 17234951 PMC1821121

[B35] Petanjek Z. Judaš M. Kostović I. Uylings H. B. (2008). Lifespan alterations of basal dendritic trees of pyramidal neurons in the human prefrontal cortex: a layer-specific pattern. Cereb. Cortex 18, 915–929. 10.1093/cercor/bhm124 17652464

[B36] Power J. D. Barnes K. A. Snyder A. Z. Schlaggar B. L. Petersen S. E. (2012). Spurious but systematic correlations in functional connectivity MRI networks arise from subject motion. Neuroimage 59, 2142–2154. 10.1016/j.neuroimage.2011.10.018 22019881 PMC3254728

[B37] Power J. D. Fair D. A. Schlaggar B. L. Petersen S. E. (2010). The development of human functional brain networks. Neuron 67, 735–748. 10.1016/j.neuron.2010.08.017 20826306 PMC2941973

[B38] Rakic P. Bourgeois J.-P. Goldman-Rakic P. S. (1994). Synaptic development of the cerebral cortex: implications for learning, memory and mental illness. Prog. Brain Res. 102, 227–243. 10.1016/s0079-6123(08)60543-9 7800815

[B39] Smith S. M. Fox P. T. Miller K. L. Glahn D. C. Fox P. M. Mackay C. E.. (2009). Correspondence of the brain’s functional architecture during activation and rest. Proc. Natl. Acad. Sci. U S A 106, 13040–13045. 10.1073/pnas.0905267106 19620724 PMC2722273

[B40] Smith S. M. Jenkinson M. Woolrich M. W. Beckmann C. F. Behrens T. E. Johansen-Berg H.. (2004). Advances in functional and structural MR image analysis and implementation as FSL. Neuroimage 23, S208–S219. 10.1016/j.neuroimage.2004.07.051 15501092

[B41] Spreng R. N. Sepulcre J. Turner G. R. Stevens W. D. Schacter D. L. (2013). Intrinsic architecture underlying the relations among the default, dorsal attention and frontoparietal control networks of the human brain. J. Cogn. Neurosci. 25, 74–86. 10.1162/jocn_a_00281 22905821 PMC3816715

[B34] Tzourio-Mazoyer N. Landeau B. Papathanassiou D. Crivello F. Etard O. Delcroix N.. (2002). Automated anatomical labeling of activations in SPM using a macroscopic anatomical parcellation of the MNI MRI single-subject brain. Neuroimage 15, 273–289. 11771995 10.1006/nimg.2001.0978

[B43] Uddin L. Q. Supekar K. Menon V. (2010). Typical and atypical development of functional human brain networks: insights from resting-state FMRI. Front. Syst. Neurosci. 4:21. 10.3389/fnsys.2010.00021 20577585 PMC2889680

[B44] Vincent J. L. Kahn I. Snyder A. Z. Raichle M. E. Buckner R. L. (2008). Evidence for a frontoparietal control system revealed by intrinsic functional connectivity. J. Neurophysiol. 100, 3328–3342. 10.1152/jn.90355.2008 18799601 PMC2604839

[B45] Vogel A. C. Power J. D. Petersen S. E. Schlaggar B. L. (2010). Development of the brain’s functional network architecture. Neuropsychol. Rev. 20, 362–375. 10.1007/s11065-010-9145-7 20976563 PMC3811138

